# Be on Target: Strategies of Targeting Alternative and Lectin Pathway Components in Complement-Mediated Diseases

**DOI:** 10.3389/fimmu.2018.01851

**Published:** 2018-08-08

**Authors:** József Dobó, Andrea Kocsis, Péter Gál

**Affiliations:** Institute of Enzymology, Research Centre for Natural Sciences, Hungarian Academy of Sciences, Budapest, Hungary

**Keywords:** complement system, lectin pathway, alternative pathway, complement inhibitors, complement-related diseases

## Abstract

The complement system has moved into the focus of drug development efforts in the last decade, since its inappropriate or uncontrolled activation has been recognized in many diseases. Some of them are primarily complement-mediated rare diseases, such as paroxysmal nocturnal hemoglobinuria, C3 glomerulonephritis, and atypical hemolytic uremic syndrome. Complement also plays a role in various multifactorial diseases that affect millions of people worldwide, such as ischemia reperfusion injury (myocardial infarction, stroke), age-related macular degeneration, and several neurodegenerative disorders. In this review, we summarize the potential advantages of targeting various complement proteins with special emphasis on the components of the lectin (LP) and the alternative pathways (AP). The serine proteases (MASP-1/2/3, factor D, factor B), which are responsible for the activation of the cascade, are straightforward targets of inhibition, but the pattern recognition molecules (mannose-binding lectin, other collectins, and ficolins), the regulatory components (factor H, factor I, properdin), and C3 are also subjects of drug development. Recent discoveries about cross-talks between the LP and AP offer new approaches for clinical intervention. Mannan-binding lectin-associated serine proteases (MASPs) are not just responsible for LP activation, but they are also indispensable for efficient AP activation. Activated MASP-3 has recently been shown to be the enzyme that continuously supplies factor D (FD) for the AP by cleaving pro-factor D (pro-FD). In this aspect, MASP-3 emerges as a novel feasible target for the regulation of AP activity. MASP-1 was shown to be required for AP activity on various surfaces, first of all on LPS of Gram-negative bacteria.

## Brief Overview of the Complement System

### Initiation Phase

**Figure 1:**
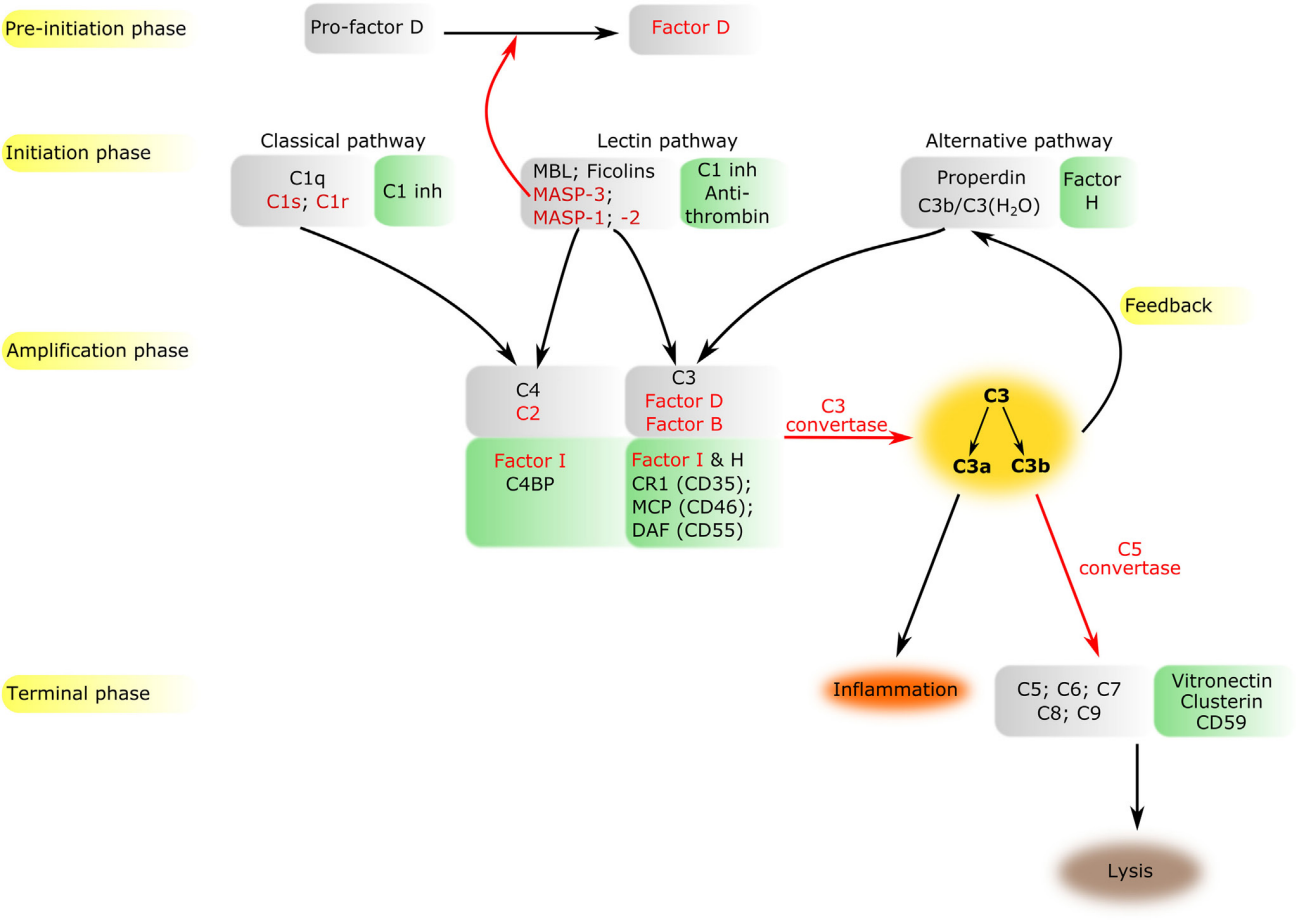
Overview of the complement system. The components of all activation phases are listed in gray boxes, while their inhibitors in green boxes nearby. Serine proteases are indicated by red letters. Black arrows indicate the direction of the cascade. Certain enzymatic cleavages are emphasized by red arrows. The three activation routes merge at the cleavage of C3 highlighted by dark yellow background.

**Figure 2:**
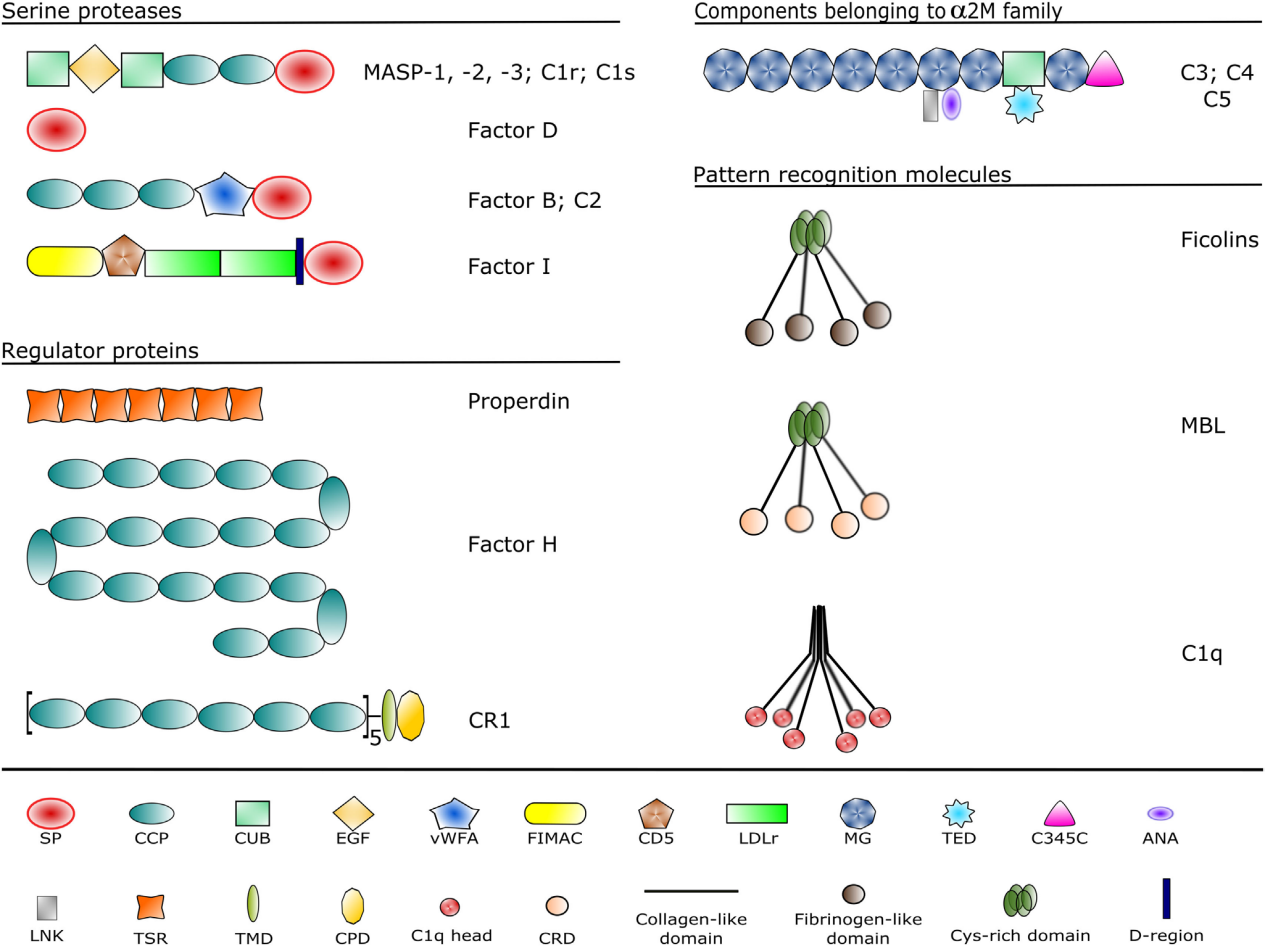
Domain structures of the complement components discussed in the article. Each domain type is represented by a different symbol listed at the bottom. Domain abbreviations are as follows: SP, serine protease; CCP, complement control protein; CUB, complement C1r/C1s, sea urchin Uegf and bone morphogenetic protein-1; EGF, epidermal growth factor-like; vWFA, von Willebrand factor type A; FIMAC, factor I/membrane attack complex; CD5, scavenger receptor cysteine-rich domain; LDLr, low-density lipoprotein receptor; MG, α-macroglobulin; TED, thioester domain; ANA, anaphylatoxin; LNK, linker; TSR, thrombospondin-type 1 repeat; TMD, transmembrane domain; CPD, cytoplasmic domain; CRD, carbohydrate recognition domain.

The complement system is a sophisticated network of serum proteins (recognition molecules, proteases, modulators, inhibitors) as well as cell-surface regulators and receptors that constitute a key part of the host defense machinery. The complement system is a powerful effector component of the innate immunity and a vital modulator of the adaptive immune response (1–3). The complement system recognizes, tags, and eliminates microbial intruders and other dangerous particles such as immune complexes, damaged, and altered self cells. The complement system is inactive (or at least shows a very low basic activity: “tickover”) until it is activated by various danger signals. There are three canonical pathways through which the complement system can be activated: the classical pathway (CP), the lectin (LP), and the alternative pathways (AP) (Figure 1). CP and LP have several features in common. In both cases, pattern recognition molecules (PRMs) bind to the danger-associated structures. The PRMs, like the other complement components, are modular proteins, consisting of multiple structural domains (Figure 2). C1q, the single PRM of the CP binds primarily to immune complexes containing IgG or IgM, and to C-reactive protein (CRP) *via* its C-terminal globular domains (4). These globular domains are fused to N-terminal collagen-like arms forming the characteristic “bunch-of-six-tulips” structure. The structure of the PRMs of the LP resembles that of C1q; globular heads and collagen-like arms. However, the recognition domains of mannose-binding lectin (MBL), other collectins, and ficolins bind to different structures. The C-type lectin domains of MBL recognize the carbohydrate pattern of the bacterial surfaces. Ficolins (ficolin 1, 2, and 3) bind to acetylated compounds, typically to acetylated sugars of bacteria, *via* their fibrinogen-like domains (5). Collectins (CL-K1 and CL-L1) also recognize sugars and other potential danger signals. Unlike C1q, which has the well-established hexamer structure, MBL, ficolins, CL-K1, and CL-L1 exist in different oligomerization states, from dimer to hexamer; the tetramer being the dominant form at least for MBL. These PRMs circulate in complex with serine protease (SP) zymogens and monitor continuously for dangerous particles the bloodstream. When the PRMs bind to the target surface, the associated SPs become activated and initiate a proteolytic cascade system, which amplifies the initial signal tremendously. C1q is associated with two C1r and two C1s proteases (the so-called “tetramer”) to form the C1 complex of the CP (6). MBL/ficolin-associated serine protease 1 and 2 (MASP-1 and MASP-2) are the initial proteases of the LP (7, 8). These SPs, together with the third MBL/ficolin-associated SP (MASP-3) form a protease family with the same domain structure (Figure 2) and similar function. The activation of the CP and LP results in the formation of the same enzyme complex, a C3 convertase (C4b2a) that cleaves C3, the central component of the complement system. The first enzymatic step in the CP activation is the autoactivation of C1r. Activated C1r then cleaves zymogen C1s, which in turn cleaves C4 and C2. In the LP, MASP-1 autoactivates first and then cleaves MASP-2 (9). MASP-2 is the enzyme of the LP that cleaves C4 (10, 11), while C2 is cleaved by both MASP-1 and MASP-2. C3 and C4 are closely related thioester-containing proteins that form the basis of the convertase complexes (12, 13). Their function is to covalently attach the convertase to the activation surface and to capture the SP components of the enzyme complex. C2 is the SP component of the C3 convertase of the CP and LP. Activation of the AP is quite different from that of the CP and LP (14). When the CP/LP C3 convertase (C4b2a) cleaves C3, a smaller fragment is released (C3a). The larger fragment (C3b) covalently binds to the activation surface preferably through an ester or, less likely, through an amide bond due to the reaction of the exposed thioester bond (15, 16). The nascent C3b component binds factor B (FB), the SP component of the AP C3 convertase. FB is cleaved by FD, a SP which circulates predominantly in its cleaved form in the blood. The resulting C3bBb is the AP C3 convertase, which converts more C3 into C3b. The new C3b molecules serve as platforms for new C3 convertase complexes. In this way, a positive feedback loop amplifies the initial signal tremendously generated either by the CP or the LP (17). According to the C3 tickover hypothesis, the AP can also initiate on its own without involvement of CP or LP (18). The circulating C3 molecules hydrolyze slowly and spontaneously in the bloodstream. The resulting C3(H_2_O) is a C3b-like molecule; it can bind FB and then form an “initiation” C3 convertase (C3(H_2_O)Bb). If this fluid-phase C3 convertase emerges near a surface, the nascent C3b molecules can bind to the surface and initiate the positive feedback loop. In this way, the AP continuously monitors the different surfaces and if it finds an activator surface, it launches efficient complement activation. The self-tissues are protected from AP-mediated damage by cell-bound and fluid-phase inhibitors (Figure 1). These inhibitors dissociate the C3bBb complex and serve as cofactors for the serine protease factor I (FI) in the degradation of C3b (19). Decay-accelerating factor (DAF, CD55), membrane cofactor protein (MCP) (CD46), complement receptor 1 (CR1, CD35) are cell-surface-bound while the master regulator of the AP is the fluid-phase protein, factor H (FH). FH binds to cell-surface-deposited C3b and facilitates its degradation to iC3b, C3c, and C3dg by FI. On endogenous cell membranes, which expose sialic acid, binding of FH is tight and the degradation of C3b is rapid, while on the so-called “protected surfaces” (e.g., bacteria, fungi) binding is weak and the amplification loop of AP can build up. There is a positive regulator of the AP, properdin, which increases the half-life of the C3bBb complex. Originally, at its discovery, properdin was regarded as a pattern recognition-like initiator molecule of the AP (20). Later, it was considered as a positive regulator (21); however, recently, the pattern recognition function of properdin has been reconsidered (22, 23).

### From the Central Phase to the Terminal Pathway

**Figure 3:**
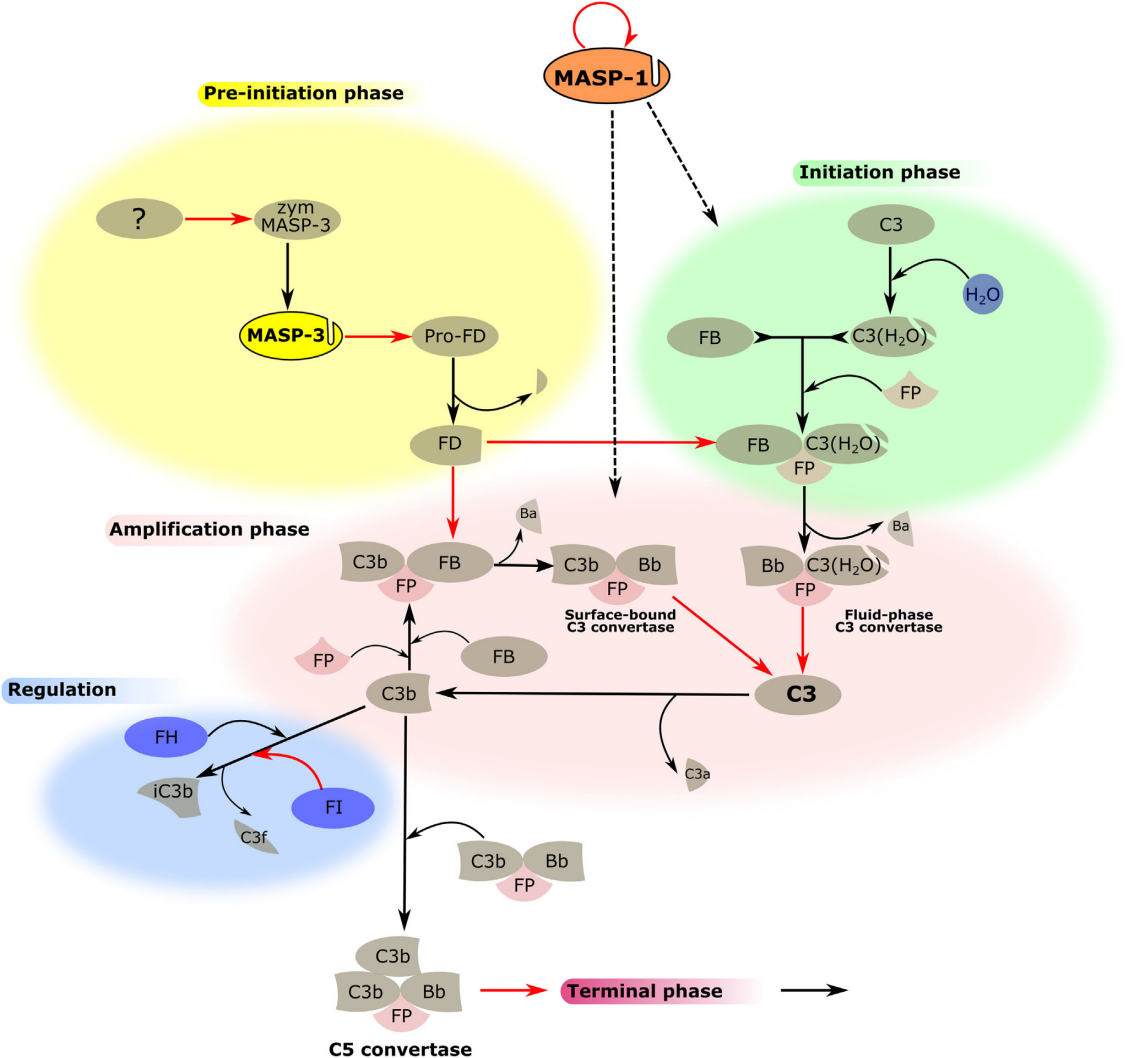
MASP-1 and MASP-3 play roles in the activation of the alternative pathway (AP). The activation process of the AP is divided into several phases, which are indicated by differently colored backgrounds. MASP-3 is proved to be the professional activator of pro-factor D in blood, therefore, plays an important role in the pre-initiation phase. The physiological activator of zymogen MASP-3 (zym MASP-3) is hitherto undiscovered (shown by the question mark). MASP-1 is indispensable for efficient initiation and amplification of the AP on certain surfaces, although the mechanism is yet unknown (shown by dashed arrows). Black arrows represent conversion processes, while red arrows stand for enzymatic reactions pointing from the enzyme toward its substrate. The circle-shaped red arrow symbolizes the autoactivation of MASP-1.

The cleavage of C3 by the C3 convertases is the turning point of complement activation. At this point, the three activation pathways (CP, LP, and AP) merge into a unified terminal pathway (Figures 1 and 3). When the density of surface-deposited C3b reaches a certain level, the substrate specificity of the C3 convertases switches to cleave the C5 component. The C4b2a(C3b)n and C3bBb(C3b)n convertases cleave the C5 component into a smaller (C5a) and larger (C5b) fragments. The C5a fragment, like the C3a fragment, is an anaphylatoxin. The anaphylatoxins bind to their receptors (C3aR and C5aR1/2) on leukocytes and endothelial cells and initiate inflammatory reactions (24). Structurally, C5 is similar to C3 and C4 (although it does not contain thioester bond) (25). The cleavage of C5 is the last enzymatic step in the complement cascade. From this point, conformational changes drive the formation of a self-organizing protein complex that damages the membrane of the attacked cells [membrane attack complex (MAC)]. After cleavage, the nascent C5b undergoes a conformational change that enables it to bind the C6 and C7 components (26). The resulting C5b–C7 complex binds to the cell membrane and captures C8. After conformational changes, C8 integrates into the membrane and pave the way to the integration of multiple copies of C9 molecules. The C9 molecules form a pore in the membrane, which results in the disintegration and destruction of the cell (27).

## Complement-Mediated Diseases

The complement system is an extremely effective cell-killing and inflammation provoking machinery. To prevent excessive activation, the complement system is kept under strict control by the different inhibitory mechanisms. A delicate equilibrium between activation and inhibition is necessary to maintain the inflammatory homeostasis in the human body. When this equilibrium is disrupted by any reason, the self-tissues can be damaged and severe disease conditions can occur. There are many clinical disorders in which uncontrolled (or sometimes the insufficient) complement activation is involved. Usually, the etiology of these diseases is complex, and the unwanted complement activation is only one of the pathological factors. However, evidences obtained by using various disease models suggest that preventing or inhibiting the pathological complement activation can be a promising therapeutic approach.

### Insufficient Complement Activation

Since the complement system provides a first line of defense against invading pathogen microorganisms, deficiency of a complement component can lead to severe infections. The consequences could be more severe during childhood, when the adaptive immune system is not developed enough. Deficiency of the initial SPs of CP and LP (C1r, C1s, MASP-2) can result in pyogenic infections (28). Deficiency of MBL is the most common immunodeficiency in humans, affecting approximately 30% of the human population (29). It predisposes to recurrent infections in infancy; however, it is not a major risk factor in the adult population. Deficiency of the alternative and the terminal pathway components can severely compromise the defense against Gram-negative bacterial infections (30). Deficiency of properdin or deficiencies in the components of MAC are associated with infections of *Neisseria* species causing meningococcal meningitis or sepsis (31–33). A very important function of the CP is the continuous removal of immune complexes and apoptotic cells. If this pathway is compromised in systemic lupus erythematosus (SLE) due to deficiency of C1q, or C4, or C1r/s, severe autoimmune reactions occur resulting in tissue injury in the kidneys.

### Excess Complement Activation

The majority of complement-related diseases are associated with overwhelming complement activation due to inappropriate control. The kidney is especially vulnerable for complement-mediated attacks. In the case of C3 glomerulopathy, C3 deposition occurs in the glomeruli without immunoglobulin deposition (34, 35). C3 deposition in this case is likely the consequence of uncontrolled AP activation. In contrast to that, in the case of membranoproliferative glomerulonephritis, CP activation elicits C3 deposition, since immunoglobulins and C1q are also deposited (36, 37). The IgA nephropathy is characterized by deposition of polymeric IgA1, which triggers complement activation through the AP and the LP (38, 39). Atypical hemolytic uremic syndrome (aHUS) is also a complement-related disease, which can lead to end-stage renal failure (37, 40). The driving force behind aHUS is the inappropriate AP activation often due to variants of (41) or autoantibodies against (42) FH, the master regulator of the AP. aHUS is a form of thrombotic microangiopathy accompanied with thrombocytopenia, hemolytic anemia, vascular damage, and thrombosis.

Another rare clinical condition associated with uncontrolled AP activation is paroxysmal nocturnal hemoglobinuria (PNH) (43, 44). In PNH patients, red blood cells are particularly prone to complement-mediated lysis due to the lack of two membrane-bound regulator proteins: DAF (CD55) and CD59. This is the consequence of the defect in glycosyl phosphatidylinositol (GPI) synthesis in the cells. GPI is responsible for anchoring various proteins to the cell membrane including these inhibitors that regulate the activation of the AP (CD55) and the formation of MAC (CD59).

Age-related macular degeneration (AMD) is a complement-related disease, which affects a large population (about 100 million AMD cases) worldwide. It is the leading cause of blindness among the elderly in the developed world (45). Genetic analyses strongly suggest that uncontrolled complement activation, especially that of the AP, plays a major role in the pathogenesis of AMD. Genetic variants of FH, C3, FB, FI, and C9 have been associated with AMD (46). In the center of the retina of AMD patients, the photoreceptor cells are gradually degraded due to a chronic inflammation, which manifests in the accumulation of immune deposits called drusen underneath the retinal pigment epithelium. The drusens (that contain activated complement components) compromise the transport of oxygen and nutrients to the photoreceptors facilitating their degeneration. Numerous attempts have been made to curb the unwanted AP activation in the eye with limited success (47). In order to efficiently influence complement activation in the eye, we have to reveal its exact mechanism, which could be different in the periphery than in the bloodstream.

Ischemia–reperfusion injury (IRI) can be considered as a severe autoimmune reaction (48), which plays a major role in a number of clinical conditions. When the blood flow in an organ stops temporarily for any reason, the deprivation of oxygen (hypoxia) induces changes in the tissues, which predisposes them for complement-mediated attack after reperfusion. The affected cells and tissues are recognized by the immune system as damaged self [damage-associated molecular pattern (DAMP)], and a complex inflammatory reaction is launched, in which the complement system plays a decisive role. IRI significantly contributes to the tissue damage in the case of myocardial infarction, stroke, transplant-induced inflammation, and it can cause a complication during coronary artery bypass graft surgery. Although the exact mechanism of complement activation in case of IRI is not fully clarified yet, a number of evidences suggest that inhibition of the LP could be therapeutically advantageous (49–53).

Artificial materials used in modern medicine, such as polymer plastics and metal alloys, can also activate the complement system and trigger inflammation (54). Nanoparticles used as contrast agents or drug carriers can also activate the complement system, sometimes causing a severe adverse reaction, called complement activation-related pseudoallergy (55, 56). In this type of hypersensitivity reaction, IgE is not involved. Liposomal drugs directly activate the complement system liberating C3a and C5a anaphylatoxins, which trigger mast cells and basophils.

If the immune system is exposed to an overwhelming amount of danger signals [pathogen-associated molecular patterns (PAMPs) or DAMPs], a systemic inflammatory reaction can occur, which could be more devastating than the original danger source. In the case of systemic inflammatory response syndrome, such as sepsis or polytrauma, the massive and systemic complement activation fuels a vicious cycle of hyperinflammatory events that can results in fatal tissue damage (57).

A growing number of evidences indicate that the complement system plays an important role in fundamental developmental processes. The lack of functional LP components (MASP-3, CL-K1, CL-L1) during embryogenesis results in the Malpuech–Michels–Mingarelli–Carnevale (3MC) syndrome that manifest in characteristic craniofacial dysmorphism and multiple other anomalies (58, 59). It was shown that an intact CP is essential for postnatal brain development. Contribution of C1, C4, and C3 was demonstrated to synaptic pruning essential for proper neuron circuit formation (60). These complement components tag synapses and mediate their elimination during a discrete window of postnatal brain development. C1q or C3 deficiency in mice results in improper central nervous system (CNS) synapse elimination. If these processes, essential during normal brain development, are pathologically upregulated during adulthood, they can contribute to the development of neurodegenerative diseases, such as Alzheimer disease and frontal temporal dementia (61). In addition to that, uncontrolled CP and LP activation in the CNS can also contribute to psychiatric disorders such as schizophrenia (62, 63).

## Cross-Talk between the AP and the LP

As described above, there are three canonical activation routes of the complement system. It is also obvious that the CP and the LP would not work efficiently without the amplification loop provided by the AP, hence, the three pathways are naturally interconnected. It is also possible that homologous proteins C4 and C3, or C2 and FB can substitute each other to a certain degree; at least *in vitro* experiments indicate a weak cross-reactivity between CP/LP an AP C3 convertase components (64). MASP-1 (65) and MASP-2 (66) have both been implicated to be able to directly cleave C3; however, the physiological relevance of these reactions is uncertain. MBL was also shown to be involved in AP activation without the requirement of C2, C4, and MASPs (67), but in the light of our recent results, the observed effect could be mediated by MASP-1 (68). In summary, the involvement of the LP or LP components in AP activation has been demonstrated in the literature before; however, some results still remain controversial. In the subsequent two sections, recent discoveries are presented regarding the role of MASP-3 during the very early stage of AP activation, and the requirement of MASP-1 for AP activation on various surfaces.

### Active MASP-3 Is the Professional Pro-FD Maturase in Blood

The first evidence that MASP-1 or MASP-3 might have an essential role in AP function came from the group of T. Fujita (69). They created *MASP1* knock-out mice by replacing the second exon (8). Since this region encodes a common part of both MASP-1 and MASP-3, the final homozygous mouse strain lacked both proteins. Surprisingly, these mice had pro-FD in their sera and had no AP activity (69). They suggested that MASP-1 acts as an essential enzyme for pro-FD maturation. At the time, it seemed like a logical assumption to favor MASP-1 over MASP-3 since MASP-1 is a more active enzyme in general with a relatively broad substrate specificity (70). Later, the same group suggested that MASP-3 might be more important than MASP-1 in pro-FD activation and suggested that even the proenzyme form of MASP-3 can act as the activator (71).

Subsequent publications questioned the requirement of either MASP-1 or MASP-3 for AP activity. In the serum of a 3MC syndrome patient lacking both proteins, functional AP was observed (72), and in mice deficient for MASP-1, MASP-3, and FH extensive, AP activation was observed, just like in mice deficient for FH only (73).

To clarify the roles of MASPs in pro-FD activation, we set up a series of experiments. *In vitro* all active MASPs were shown to be able to cleave pro-FD efficiently to produce FD, whereas the MASP zymogens lacked such activity (74). We prepared fluorescently labeled pro-FD, added it to different types of human plasma and serum preparations and followed the conversion of pro-FD to FD. Pro-FD was efficiently cleaved in all types of blood preparations, even in citrated and EDTA plasma, where neither the complement nor the coagulation cascade is expected to be activated. This experiment established that at least one protease is present in normal human blood capable of converting pro-D to FD without the prior activation of the abovementioned proteolytic cascades. Using a MASP-1-specific and a MASP-2-selective inhibitor, these two enzymes could be excluded. After adding recombinant active catalytic fragments of MASPs to normal human plasma samples, the half-life of labeled pro-FD was markedly reduced upon the addition MASP-3, whereas the other two enzymes had no effect (74).

The final “killer” experiment that established MASP-3 as the professional (near exclusive) activator of pro-FD came using a MASP-3 specific inhibitor, TFMI-3 (75). TFMI-3 blocked the conversion of labeled pro-FD to FD in citrated plasma, EDTA plasma, and hirudin plasma completely, while in serum, the half-life was markedly increased. Another conclusion of our studies was that active MASP-3 must be present in the blood, since only the activated form of MASP-3 can convert pro-FD to FD. Later, we provided direct evidence for the extensive basal-level activation of MASP-3 in human blood by an unknown mechanism (76). Finally, the debate seems to have settled. A recent paper showed that in 3MC syndrome patients lacking MASP-3, predominantly pro-FD is present in their sera, and moreover, in healthy individuals, some pro-FD is also present beside the dominant active form (77).

The picture is now clear. Under normal circumstances, active MASP-3 is present in the blood, which activates pro-FD, therefore, continuously supplying active FD for the AP (Figure 3). However, MASP-3-deficient individuals are not completely defenseless. At least one coagulation enzyme can probably also provide low levels of FD for the AP, or when the LP is activated, MASP-1 or MASP-2 might also contribute. These backup mechanisms also need some consideration when targeting MASP-3 to control AP activity, as it will be discussed later.

### MASP-1 Is Required for AP Initiation on Certain Activating Surfaces

Despite the fact that molecular mechanisms of the complement system have been thoroughly examined in the past decades, still many questions remained about the early steps of the activation. MASP-1 as a promiscuous enzyme with broad substrate specificity (70) has the potential to replace other SPs or amplify enzymatic reactions. It has been reported that MASP-1 is indeed involved in biological processes beyond LP or even the complement system.

Recently, we have found a novel function of MASP-1 in AP activation apart from its role in the LP (Figure 3). This function suggests an unexpected linkage between the two pathways and also highlights the differences between various activation surfaces (68). Previously, specific and highly selective small-protein inhibitors against all MASPs were developed from canonical inhibitor scaffolds using the phage-display technique. SGMI-1 is a specific MASP-1 inhibitor, whereas SGMI-2 inhibits MASP-2, and TFMI-3 is a specific inhibitor of MASP-3 (9, 75). Activation of the AP *in vitro* can be carried out on ELISA microtiter plates coated by bacterial lipopolysaccharide (LPS) or yeast zymosan. These materials serve as models of the pathogenic surfaces. In the absence of Ca^2+^ using Mg^2+^-EGTA buffer, AP activation can be initiated without the involvement of CP and LP. Administration of the specific MASP-1 inhibitor, SGMI-1 in this system lead to surprising results. The activity of the AP was attenuated significantly through the inhibition of MASP-1. However, this effect was only seen on the bacterial surface represented by LPS, while zymosan-induced AP activation was not compromised. To rule out the possibility that SGMI-1 may impede other SPs, inhibitors of MASP-1 possessing different mode of action were also tested. Anti-MASP-1-SP antibody, N-terminal domains of MASP-1 (M1_D1-3) (78), and serpin domain of C1 inhibitor resulted in the same, considerable reduction of AP activity but only on LPS. The activity of AP in MASP-1-depleted serum remarkably decreased on LPS-coated surface while on zymosan-coated surface, it was only moderately affected. The mechanism of C3b deposition, which is followed in our assay, can be divided to initiation and amplification phases. Time-course measurement of C3b deposition in the presence of subsequently added SGMI-1 inhibitor indicated that MASP-1 contributes to both phases of C3b generation. Although we proved unambiguously that MASP-1 has an effect on AP activation, there are still many puzzling details to be solved. First, we tested the known components of AP as possible reaction partners of MASP-1. It was clarified earlier that MASP-1 cleaves C3 only at a very low rate (11) and now we confirmed that MASP-1 does not react significantly with C3b-bound FB. The contribution of FD was also excluded since SGMI-1 does not inhibit FD, and MASP-1 is not the physiological activator of pro-FD (74). Our results lead to the conclusion that the key player of MASP-1-driven AP activation is probably not among the core components of the AP.

Differences between the activating surfaces also need further investigations since it seems to be likely that AP initiation occurs by various mechanisms. Using specific antibodies in the ELISA system, neither MASP-1 nor MBL could be detected on LPS surface in Mg^2+^-EGTA buffer (68). MASP-1 may be presented by some other PRMs, which do not necessarily require Ca^2+^ for binding (possibly ficolins). Another possible scenario is that MASP-1 forms a labile and transient complex with its reaction partners. One clue arises from the literature that properdin, which stabilizes C3bBb complex, is crucial for LPS-induced but not for zymosan-induced AP activation (79). Another coincidence is that LPS, rather than zymosan, binds FH with high affinity, which enhances the decay of C3 by FI activity. The ratio of these factors, which play a role in the regulation of AP, may be influenced by MASP-1 through a yet unknown mechanism.

These new findings draw attention to MASP-1 in the promotion of LPS-induced AP and, therefore, its role in the defense against Gram-negative bacteria.

## Components of AP and LP as Potential Drug Targets

### Pattern Recognition Molecules

The PRM of the CP and LP recognize danger signals (PAMPs and DAMPs) and provide the framework of the initiation multimolecular complexes (the C1 complex, MBL–MASP complexes, ficolin–MASP complexes). They have similar overall domain architecture: N-terminal collagen-like domains and C-terminal globular domains. The structure of C1q is different from that of the other PRMs, since the basic trimeric subunit of C1q is composed of three different polypeptide chains (A, B, and C chains), while that of MBL and ficolins consist of only one kind of polypeptide chain. In the case of collectins, collectin kidney 1 (CL-K1) and collectin liver 1 (CL-L1), a heterotrimeric subunit was observed in human blood composed of one CL-L1 and two CL-K1 polypeptide chains (called CL-LK) (80). Another structural difference between C1q and the proteins of the collectin/ficolin family is that the latter contain a short N-terminal cysteine-rich region and an α-helical coiled-coil neck region between the collagen-like and the globular domains facilitating trimerization of the polypeptide chains. The collagen sequences (Gly-Xaa-Yaa repeats) are interrupted at one point in C1q and MBL generating a flexible kink region that may play an important role in binding to the danger patterns and activating the associated SPs. The PRMs bind the associated SPs in a Ca^2+^-dependent manner. C1q binds the C1s-C1r-C1r-C1s tetramer, while MBL, ficolins, and CL-LK bind MASP dimers. It is probable that low oligomeric MBL and ficolins bind a single MASP dimer, while higher oligomers (pentamers, hexamers) can bind two MASP dimers simultaneously (81). There is a cross-interaction between the components of the CP and LP; MBL can bind the C1r_2_s_2_ tetramer and C1q can bind the MASP dimers, although with reduced affinity compared to the cognate pairs (82). It is unlikely that these interactions have a physiological relevance (except maybe in deficiencies); however, the existence of these cross-bindings proves that the interactions are analogous between the PRMs and the associated SPs among the components of the CP and LP. The binding of MBL and ficolins to their targets is Ca^2+^-dependent. The affinity of a single carbohydrate-binding domain to its target sugar is low (Kd in the millimolar range), whereas the avidity of the whole oligomeric molecule is high with a Kd in the low nanomolar range. It is very likely that the PRMs of higher oligomeric state activate the LP more efficiently than the low oligomeric PRMs due to the stronger binding to both the target surface and to the MASPs (83). The mechanism of activation of the C1 and MBL–MASP complexes is not fully clarified yet.

Theoretically, if we want to prevent or inhibit improper complement activation in a pathological situation PRMs of the initiation complexes are ideal targets; since by inhibiting the PRMs, we can shut off the entire amplification machinery of the complement system at the very first step. There are three possibilities to inhibit the function of the PRMs: (1) to prevent the binding of the PRMs to their target; (2) to prevent the binding of the associated SPs to the PRMs; (3) to prevent the conformational changes of the PRMs that are necessary for the activation of the SPs. Monoclonal antibodies that bind to the globular domains of the PRMs can efficiently interfere with the ligand binding. Anti-C1q and anti-MBL antibodies were successfully used to block the CP and LP activation, respectively (84). An anti-MBL monoclonal antibody (3F8) attenuated myocardial IRI in mouse expressing human MBL (85).

Anti-C1q antibodies have been recently reported to greatly reduce the inflammatory demyelinating lesions in a mouse model of neuromyelitis optica (86) and also to attenuate injury with a consequent neuroprotective effect in acute Guillain–Barré syndrome mouse models (87). A peptide agent (called 2J) was selected from a peptide library on the basis of C1q binding (88). This peptide was shown to bind to the globular domain of C1q and prevented the binding of C1q to IgG. The 2J peptide efficiently inhibited CP-mediated C4 and C3 deposition and MAC formation *in vitro*. Although this peptide was a promising candidate for therapeutic complement inhibition, no further studies were reported about its *in vivo* application.

Another possibility for inhibiting the CP and the LP is to disassemble the initiation complexes. In this respect, it is worth noting that in *in vitro* experiments the C1 complex dissociates in the absence of Ca^2+^ (in the presence of EDTA), or at high ionic strength (1 M NaCl), whereas in the case of the MBL–MASP complexes, both conditions should apply at the same time (89). Moreover, C1 inhibitor, which makes covalent complexes with the SPs, dislodges C1r and C1s from C1q, while it cannot disassemble the MBL–MASP complexes (90). Nevertheless, it was shown that there is a dynamic equilibrium between the different MBL/ficolin–MASP complexes in human serum, in other words, MASPs can migrate between the complexes (91). Recently, it was shown that asparaginase, which is used in oncological treatments, inhibits the LP by reducing the amount of MBL–MASP complexes, very likely through dissociating the complexes (92). In this case, it is an adverse effect of the oncological treatment, but it indicates that a similar approach can be feasible in anticomplement therapy. An anti-MASP-2 monoclonal antibody (OMS721, Omeros), which binds to a non-catalytic complement control protein (CCP) domain of MASP-2, successfully inhibited the LP in *in vivo* experiments, and also it could disassemble the MBL-MASP-2 complexes.

There is a report about a viral-derived peptide (PIC1), which inhibits the classical pathway through binding to the collagen-like region of C1q in the C1 complex (93). This peptide might lock the conformation of C1q and/or displace the tetramer. There is no other report in the literature about an agent, which can block the conformational change necessary for the proper function of the initiation complexes. A deeper understanding of the activation mechanism of the C1 and MBL/ficolin–MASP complexes is needed to harness this possibility in the therapy.

### MASP-1

MASP-1 is the most abundant protease of the LP, and it plays a central role in complement activation. Its average serum concentration is 143 nM (11 µg/mL), which is 24-times higher than the serum concentration of MASP-2 (6 nM, 0.4 µg/mL) (94). The members of the C1r/C1s/MASP protease family share the same domain organization (Figure 2). At the N-terminus, there is a CUB domain (initially recognized in *C*1r/C1s, sea urchin protein *U*egf, and human *b*one morphogenetic protein 1), followed by an epidermal growth factor-like (EGF) module and a second CUB domain. The MASPs are present as dimers in the circulation, and the N-terminal CUB1-EGF-CUB2 region is responsible for the dimerization. Another important function of this region is that it mediates the binding to the PRMs. The CUB and the EGF domains bind Ca^2+^, and both the dimerization and the PRM binding are Ca^2+^-dependent. The C-terminal region, which possesses the catalytic activity, consists of two CCP domains and a SP domain. The SP domain belongs to the chymotrypsin family (Family S1, MEROPS) and shows trypsin-like specificity cleaving after basic amino acids (Arg, Lys) in the polypeptide chain. The two CCP domains have at least two functions: they serve as spacers between the CUB1-EGF-CUB2 region and the SP domain and they provide additional binding sites (exosites) for the substrates (13). Both functions have essential roles in the activation of the PRM–MASP complexes and in the cleavage of the subsequent components (C2 and C4).

MASP-1 has multiple roles in the innate immune response. Zymogen MASP-1 has a high autoactivation capacity, which plays a key role in the activation of the lectin pathway (95). When PRM–MASP complexes bind to the activation surface, zymogen MASP-1 autoactivates and the active MASP-1 activates zymogen MASP-2 (9, 72). In this way, MASP-1 is the initiator protease of the LP. Recently, it has been demonstrated that MASP-1 significantly contributes to AP activation on LPS surface through an unknown mechanism (68). MASP-1 is also capable of activating endothelial cells by cleaving protease-activating receptor 4 (91, 96). The activated endothelial cells secrete cytokines (IL-6 and IL-8), and these cytokines promote the chemotaxis of neutrophil granulocytes (97). Moreover, MASP-1 treatment increased adhesion between neutrophils and endothelial cells by upregulating E-selectin expression in human umbilical vein endothelial cells (HUVECs) (98). A genome-wide gene expression profiling study on HUVECs corroborated the role of MASP-1 in triggering inflammation (99). The analysis showed that MASP-1 up- and downregulated numerous inflammation-related genes bridging complement activation and endothelial-cell-related inflammatory processes. It was also demonstrated that MASP-1 is able to cleave high-molecular-weight kininogen and liberate bradykinin (100). Bradykinin is a potent vasoactive, pro-inflammatory peptide, which is responsible for the swelling attacks in hereditary angioedema (HAE), a disease associated with C1 inhibitor deficiency (101). Uncontrolled activation of MASP-1 may contribute to the development of HAE attacks and worsening the symptoms of HAE patients. It was also recognized that MASP-1 serves as a link between the complement and the coagulation cascades. MASP-1 promotes coagulation by activating prothrombin, fXIII, and thrombin-activatable fibrinolysis inhibitor (102–104). The effect of MASP-1 on blood coagulation was confirmed by using a microvascular whole-blood-flow model (105). The physiological relevance of this phenomenon is not quite clear; however, it is very likely that the proteolytic activity of MASP-1 contributes to pro-inflammatory and pro-thrombotic events facilitating the development of thrombotic complications under pathological conditions (106). As the above examples highlight, MASP-1 has a relatively broad substrate specificity (it has about 10 known substrates), which is quite unusual among complement proteases. It should be noted, however, that all the known substrates of MASP-1 are related to the innate immune response. Evolutionary considerations indicate that MASP-1 is an ancient enzyme of the complement system compared to the other members of the MASP/C1r/C1s family (107). The relaxed substrate specificity of MASP-1 is reflected in its 3D structure (70). The substrate-binding groove of MASP-1 is broad and accessible resembling that of trypsin, rather than those of other early complement proteases. The physiological inhibitors of MASP-1 are serpins. C1 inhibitor, and in the presence of heparin antithrombin, attenuate very efficiently the activity of MASP-1 (108). Alpha_2_-macroglubulin, a pan-specific protease inhibitor in the blood was suggested to inhibit MASP-1 and consequently the LP (109), but this issue is controversial (108, 110). Another potential physiological inhibitor of the LP is MAp44 (aka MAP-1), an alternative splice product of the *MASP1* gene (111, 112). MAp44 contains the CUB1-EGF-CUB2-CCP1 domains of MASP-1/3 plus a 17 amino-acid-long C-terminal peptide. Since MAp44 lacks the SP domain, it does not have proteolytic activity to initiate the LP, but it can dimerize and bind to the PRMs like the MASPs. MAp44 attenuates LP activity by competing with MASP-1 and MASP-2 for the PRMs and displacing them from the complexes. Recombinant MAp44 was shown to protect against myocardial IRI in mouse models, preserving cardiac function, decreasing the infarct size, and preventing thrombogenesis (113). Recombinant chimeric inhibitors were also designed and constructed by fusing MAp44 and the complement regulatory domains (1–5) of FH (114). One of these inhibitors showed simultaneous inhibition of the LP and AP.

**Figure 4:**
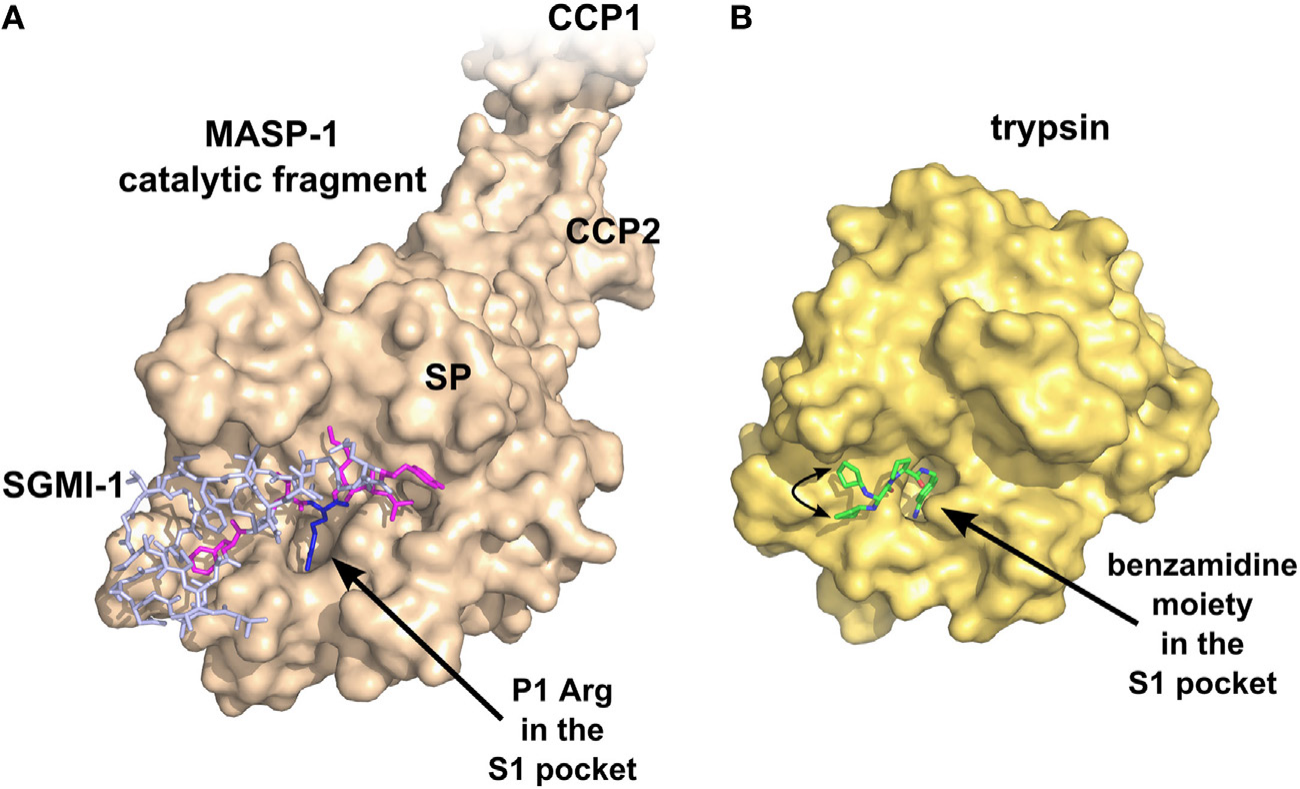
Specific inhibition of proteases requires multiple favorable contacts in a large contact area with an inhibitor. **(A)** The structure of MASP-1 in complex with a specific small-protein inhibitor, SGMI-1 (PDB entry 4DJZ). SGMI-1 was developed by phage-display (122). Amino acid residues in the randomized positions are colored magenta (P4, P2, P1′, P2′, P4′) and blue (P1). All amino acid residues in the randomized positions have contacts with the protease body; moreover, SGMI-1 has other contact areas with MASP-1 in the non-randomized positions as well. **(B)** The structure of trypsin in complex with a non-specific small-molecule inhibitor (123) (PDB entry 3LJJ). The inhibitor is based on benzamidine. A two-headed arrow indicates the movement of the terminal cyclopentane moiety, which has two equivalent binding sites.

**Figure 5:**
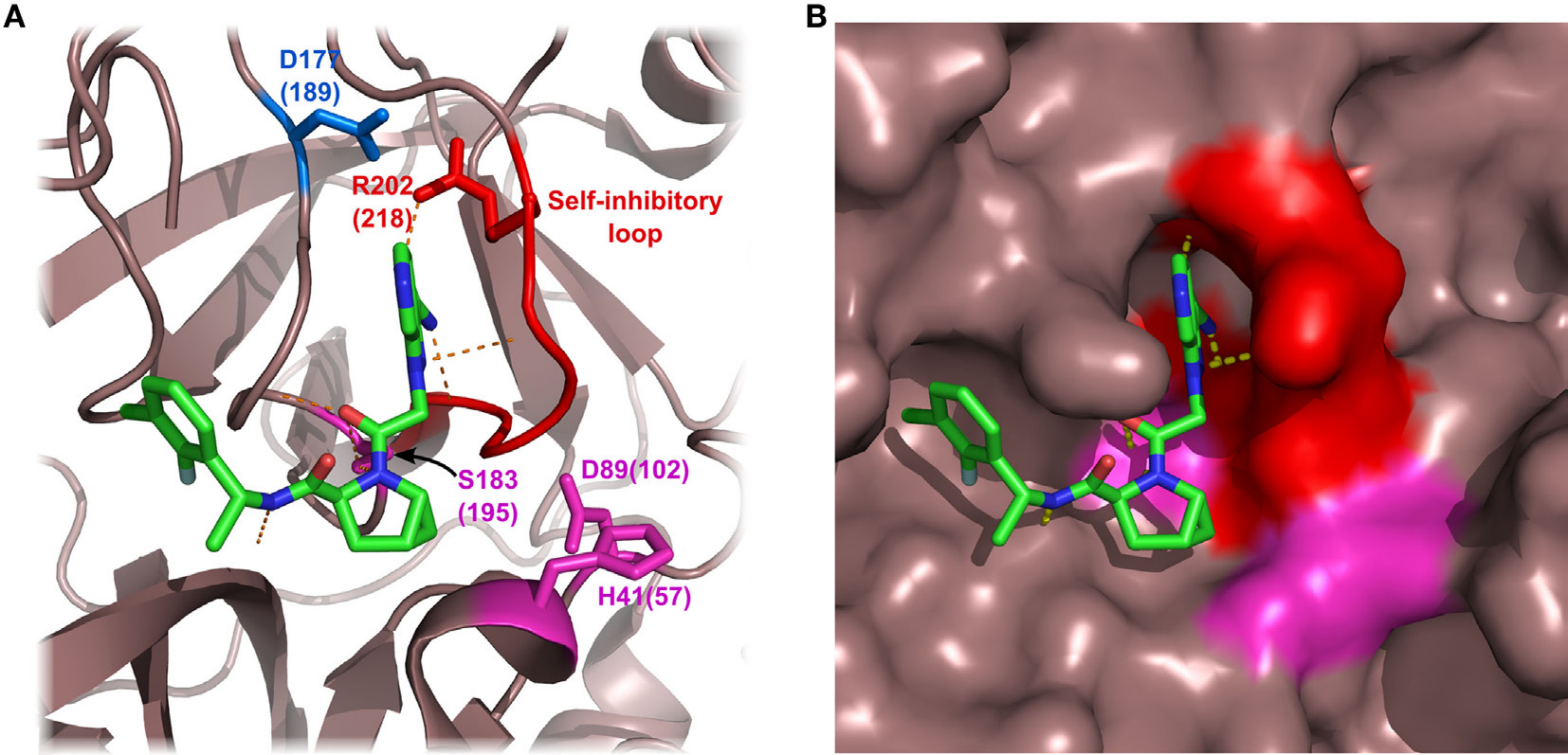
Structure of factor D (FD) in complex with a selective small-molecule inhibitor. The figure is based on the structure of FD in complex with “inhibitor 6” described by Maibaum et al. (120) (PDB entry 5FCK). Inhibitor 6 has multiple polar and hydrophobic interactions with the protein body. It is notable that inhibitor 6 interacts with the self-inhibited conformation of FD, probably stabilizing FD in this form. Asp^177^ (blue) of the S1 pocket forms a salt bridge with Arg^202^ of the self-inhibitory loop (red). The catalytic triad is colored magenta. Numbers indicate amino acid positions in mature FD, while numbers in parenthesis reflect the traditional chymotrypsinogen numbering. Hydrogen bonds are indicated by yellow dashed lines. **(A)** FD shown by ribbon representation. **(B)** FD shown by surface representation.

Theoretically, the SPs are the most druggable targets in the complement system (115). The active sites of these enzymes can be easily targeted by small-molecule protease inhibitors. The main problem with this approach is the lack of specificity, since all the complement proteases and also the proteases of the other plasma cascade systems contain chymotrypsin-like SP domains (Figure 4). A small-molecule SP inhibitor, which blocks the activity of a particular complement SP, very likely will inhibit other complement proteases, as well as proteases of the coagulation, fibrinolysis, and kallikrein–kinin systems to some extent. For example, nafamostat mesilate (FUT-175 or Futhan) is a powerful inhibitor of the complement cascade, but it has a broad specificity. It was shown to attenuate renal and myocardial IRI (116, 117), but it also attenuates pancreatitis by inhibiting trypsin and other pancreatic enzymes (118), and also coagulation by inhibiting thrombin and other clotting enzymes (119). To enhance the specificity, the number of interactions should be increased between the SP and the inhibitor. A promising approach could be the fragment-based drug discovery, which generates highly specific molecules *via* linking small chemical fragments (Mw < 300 Da) together that bind only weakly on their own to the target. This approach was successfully used to develop specific small-molecule inhibitors against FD (120) (Figure 5), but there is no report about similar molecules against MASPs. Monoclonal antibodies and other biologics can also meet the specificity criterion. Highly selective MASP inhibitors were developed by the *in vitro* evolution of the interacting loop of canonical SP inhibitors. Sunflower trypsin inhibitor (SFTI) is a 14-amino acid-long cyclic peptide, which mimics the protease-interacting loop of the inhibitor scaffold of the Bowman–Birk inhibitor family. SFMI-1, an LP-selective peptide inhibitor was developed by phage-display selection of SFTI variants using MASP-1 as target (121). SFMI-1 proved to be a strong MASP-1 inhibitor (*K*_i_ = 65 nM), and a weak MASP-2 inhibitor (*K*_i_ = 1,030 nM). In order to further increase the specificity, a larger inhibitor scaffold was used in the phage-display selection. SGPI-2 (*S. gregaria* protease inhibitor-2) is a single domain small-protein inhibitor (35-amino acid-long) belonging to the Pacifastin family of canonical inhibitors. After randomizing six positions in the protease-interacting loop (P4, P2, P1, P1′, P2′, and P4′), a highly specific MASP-1 inhibitor (SGMI-1) was selected (122) (Figure 4). SGMI-1 inhibits MASP-1 very effectively (*K*_i_ = 7 nM), and very selectively. This inhibitor was used to clarify the function of MASP-1 in the innate immune response using numerous *in vitro* and *ex vivo* assays. Although MASP-1 is a tempting target to halt unwanted LP activation and to prevent various pro-inflammatory processes, no pharmaceutical development of a MASP-1 inhibitor has been reported to date.

### MASP-2

MASP-2 is the only protease in the LP that can cleave C4. Its serum concentration is rather low (6 nM, 0.4 µg/mL), compared to the other complement proteases. These characteristics make MASP-2 an ideal target to inhibit pathological LP activation.

MASP-2 has identical domain organization with MASP-1 and MASP-3 (Figure 2). Isolated MASP-2 has a tendency to autoactivate in a concentrated solution (11, 124). This autoactivation capacity, however, cannot manifest in normal human serum, where the MASP-2 concentration is low, and each MASP-2 molecule is surrounded by MASP-1 molecules on the target surface. Under these circumstances, MASP-1 is the exclusive activator of MASP-2. The autoactivation ability of MASP-2 might be important in situations, where there is no MASP-1 present (e.g., MASP-1 deficiency). It should be noted, however, that in the serum of a 3MC syndrome patient, where there was neither MASP-1 nor MASP-3 present due to a mutation in the *MASP1* gene, no LP activity could be detected (72). On the other hand, birds lack MASP-1, but have functional LP, suggesting that MASP-2 can independently drive LP activation (125). In the sera of these animals, however, the autoactivation capacity of MASP-2 must be much higher than that of human MASP-2 in normal human serum. A recent publication shows that MASP-2 can directly cleave C3 in the absence of C4 and/or C2 on LP-activating surfaces (66). MASP-2 was also suggested to promote fibrin polymerization by cleaving prothrombin (126). The *MASP2* gene, like the *MASP1* gene, has an alternative splice product MAp19 (aka sMAP, MAP-2) (127, 128). This truncated gene product contains only the CUB1 and EGF domains plus 4 unique C-terminal residues. Since MAp19 can bind to the PRMs, it may regulate LP activity through displacing the MASPs from the complexes. Theoretically, the recombinant form of MAp19 could be suitable to attenuate LP activity, in practice, the larger MAp44 was used for this purpose since it binds to the PRMs with higher affinity.

The pathological relevance of MASP-2 was demonstrated in MASP-2 knock-out mice, where the animals were significantly protected against myocardial and gastrointestinal IRI (50). In the hearts of MASP-2-deficient mice, the infarct volume was significantly smaller than in those of the wild-type animals. Moreover, a recent study demonstrated that an inhibitory monoclonal anti-MASP-2 antibody successfully attenuated myocardial IRI in wild-type mice (129). An anti-MASP-2 antibody, OMS721, developed by Omeros Corporation, is under clinical trial for treating aHUS (130) and other thrombotic microangiopathies (131), IgA nephropathy, lupus nephritis, membranous nephropathy, and C3 glomerulopathy (132). The mechanism of the protecting effect of MASP-2 inhibition in these diseases is not clear, since AP activation is believed to be the main driver of these conditions. Selective canonical inhibitors against MASP-2 were also selected by phage-display using the SFTI and SGPI scaffolds (121, 122). Both inhibitors were highly specific: SFMI-2 (*K*_i_ = 180 nM) and SGMI-2 (*K*_i_ = 6 nM) prevented LP activation efficiently, while they did not compromise the activity of the other two pathways.

### MASP-3

MASP-3 was discovered as the third SP component of the LP (133). It has the same domain organization (Figure 2) as MASP-1 and MASP-2, as described above; moreover, the amino acid sequence of its first five domains is identical with that of MASP-1. This feature is the consequence of the fact that MASP-1 and MASP-3 are the alternative splice products of the same *MASP1* gene, along with a third protein MAp44 (111, 112). Variants of the *MASP1* gene, resulting in the loss of the activity of MASP-3, cause the 3MC syndrome, characterized by serious craniofacial, genital, and often mental defects (58, 59, 134). The results indicate that MASP-3 is involved in neural crest cell migration in early embryonic development. Interestingly, the same phenotype is observed in patients carrying mutations in the *COLEC11* gene (58) or the *COLEC10* gene (135), both encoding LP components CL-K1 (aka collectin-11) and CL-L1 (aka collectin-10). It is possible that MASP-3 is in complex with CL-K1/L1 when it exerts its function during embryogenesis, and it is likely that the proteolytic activity of MASP-3 plays an important role.

MASP-3 is different from MASP-1 and MASP-2 in several ways. MASP-3 does not autoactivate, does not cleave downstream LP/CP components, C4 and C2, and the active form has very low activity on most synthetic substrates (136). *In vitro*, it was shown to cleave insulin-like growth factor-binding protein 5; however, the relevance of this reaction is uncertain (137). It has also no natural inhibitor in the blood; therefore, control of its activity is probably achieved simply by its very restricted substrate specificity. MASP-3 is present in the blood as the mixture of the proenzymic and the activated forms; moreover, the activated form seems to be the more dominant variant (76). In this aspect, it also differs from MASP-1 and MASP-2, which are proenzymic. On the other hand, in many regards, MASP-3 has similarities to FD, which circulates predominantly in the active form, has no natural inhibitor, and has very restricted substrate specificity.

The function of MASP-3 in the blood had been mysterious until recently. Initially, it was considered simply as a negative regulator of the LP since it competes with MASP-1 and MASP-2 for binding to PRMs (133). This function may still be valid; however, now, strong evidences exist that the active form of MASP-3 is the primary physiological activator of pro-FD, producing FD, a key enzyme of the AP. The story was detailed in a previous section; therefore, we jump to the functional consequences of this activity.

It seems logical that the activity of the AP can be downregulated by the inhibition of MASP-3. Inhibition of MASP-3 would result in the accumulation of pro-FD in the blood with only very low levels of active FD, hence greatly attenuating AP activity. A study presented at 16th European Meeting on Complement in Human Disease (138) provided a strong evidence for this assumption. A single dose of a monoclonal antibody inhibiting the activity of MASP-3 suppressed the activity of the AP and shifted the active to zymogen ratio of FD toward the proenzyme, pro-FD, both in mice and in cynomolgus monkey. So far, two specific inhibitors against MASP-3 were developed. One of them is a canonical Kunitz-type recombinant protein, which is based on the second domain of tissue factor pathway inhibitor (TFPI) and developed by phage-display (75). The other is the abovementioned monoclonal antibody by Omeros Corporation (138).

What are the potential advantages of MASP-3 inhibition over FD inhibition? Inhibition of both proteins is expected to result in similar systemic effects in the blood. The plasma concentration of both proteins is similar, around 60 nM. This relatively low value is attractive for drug development. On the other hand, FD has a very high turnover rate. Its half-life in humans is less than 1 h (139). The turnover rate of MASP-3 is not yet known, but because of its size, it is most certainly lower compared to FD. This could mean that a lower daily dose of a drug candidate inhibitor of MASP-3 would be required compared to a FD inhibitor.

Deficiency in the AP can result in potentially life-threatening meningococcal infections, and AP inhibition carries the same risk. Another potential benefit of MASP-3 inhibition would be that in this case, a pro-FD pool is still available. In case of a bacterial infection, the LP can be activated, and the resulting active MASP-1 and MASP-2 molecules could locally convert pro-FD to FD, making the AP amplification possible. Nonetheless, this mechanism needs experimental validation.

In all, based on MASP-3’s requirement for the maturation of pro-FD, MASP-3 presents itself as a good target to attenuate the complement system, with several potential benefits over FD inhibition.

### Factor D

Factor D (FD) is a single domain SP, which circulates in the blood predominantly in the active form (77, 140). It is synthesized mainly by adipocytes, hence the alternative name adipsin. In the 1970s, it was debated whether it is produced as a proenzyme or secreted in the active form (140, 141). Since only the active form could be isolated from blood (142), it was assumed that it might be activated even before secretion (143). Nevertheless, at the DNA level, after the signal sequence, an additional 5 to 7 amino acid long propeptide is encoded. Now the consensus is that active MASP-3 converts the pro from of FD to the active form constitutively (74, 75, 77).

Although it is an active SP, FD has an extremely restricted substrate specificity. It has very low activity toward synthetic substrates, basically, it cleaves only certain thioester compounds; however, its natural substrate, FB in complex with C3b or C3b-like molecules, is cleaved very efficiently (144). The free enzyme’s very low activity is due to a unique self-inhibitory loop (145), which is displaced when FD binds to C3bB (146).

It has a relatively low mass concentration of 1–4 µg/mL in humans (147–149), which in combination with early reports showing that FD is the bottleneck of AP activity (140), led to the assumption that FD could be the best target to achieve AP inhibition. However, FD is a small protein of only 25 kDa, so, its molar concentration of 40–160 nM combined with its high turnover (139) suggest that high daily doses of a FD inhibitor would be required to achieve complete sustained inhibition. Recent results also suggest that FD may not even be the bottleneck of AP activity. In the serum of FD-deficient mice, the addition of FD corresponding to only about 1–2% of the normal FD level was sufficient for normal AP activity *in vitro* (147). In a 3MC syndrome patient, whose serum contained mostly pro-FD, some AP activity was still present (72), although lower than the normal level (134). These data together suggest that even if a potent and specific inhibitor is used, at least equimolar amount is required for AP inhibition, and even higher doses are necessary for sustained inhibition. A study with lampalizumab, a humanized monoclonal anti-FD IgG F_ab_ fragment, showed similar observations (150).

One must also consider that, at least *in vitro*, plasma kallikrein was shown to be able to cleave the C3bB pro-convertase (151), hence a residual, low-level AP activity might still be present even during complete FD inhibition, or FD deficiency; however, the *in vivo* relevance of this cleavage needs further validation.

Nevertheless, FD remains a prime target within the complement system. Several FD inhibitor molecules are under development, or in the clinical trial phase (152, 153) for PNH, aHUS, and AMD. Achillion developed several small molecule FD inhibitors that may be orally administered. A dose of 200 mg/kg of ACH-4471 per every 12 h resulted in complete AP inhibition in primates (154). An example of the combination of structure-based and fragment-based drug development targeting FD was published recently. Modifying the structure of a small-molecule kallikrein inhibitor several compounds were developed that selectively inhibit FD (120). Figure 5 shows FD in complex with one of the compounds as an example.

Near complete inhibition of FD is expected to have a similar outcome as inhibition of MASP-3. Neisserial infection or other bacterial infections constitute a possible threat, which requires prophylactic treatment or treatment with antibiotics. This is actually valid for nearly all kinds of anticomplement drugs.

### Factor B

Factor B (FB), a five-domain, 90 kDa glycoprotein, is composed of three CCP modules, a short connecting segment, a von Willebrand factor type A (vWFA) domain, and an SP domain (Figure 2). It circulates as a proenzyme, and its activation site (Arg^234^-Lys^235^) is hindered in the free enzyme from the cleavage by FD. FB can form a complex with C3b, or C3b-like molecules, to generate the AP pro-convertase, C3bB. The pro-convertase probably exists in two, closed and open conformations, in the latter, FB being accessible for FD cleavage (155). The FD-C3bB interaction facilitates both a shift toward the open conformation of C3bB, and a structural rearrangement in FD displacing its self-inhibitory loop (146). FD cleaves FB in the pro-convertase to release the Ba fragment. The other fragment, the catalytic Bb itself is still just a marginally active enzyme (156), it has full activity only as part of labile C3bBb complex (157). Once Bb dissociates from the convertase complex, it cannot re-associate with C3b (157).

FB is absolutely essential for the AP; therefore, it is a prime target for AP inhibition, but because of its high concentration (about 200–250 µg/mL, or 2–3 µM), it might not seem to be ideal at first sight. On the other hand, in order to prevent AP activation, only the newly formed C3bBb complexes may have to be inhibited. Using a potent inhibitor with low Kd toward C3bBb could completely block the amplification phase, thereby halting the activation process. While C3bBb might be a difficult target for testing small-molecule inhibitors, because of the transient nature of this complex, the cobra venom factor (CVF)-Bb complex is more stable; therefore, it presents itself as a viable target for the development of such molecules. It is also notable that at high pH (proenzymic), FB alone has significant, easily detectable activity toward C3 and certain para-nitroanilide substrates (158). Several substrate-analog aldehyde FB inhibitors were developed along the way (158).

Inhibitory antibodies might be more easily obtained. They only need to prevent access to C3, the very large substrate of C3bBb, which is attainable by a bulky antibody molecule binding near the catalytic site. However, it is possible that such antibody would also bind to free FB; therefore, higher doses might be required. Optimally, a small-molecule inhibitor or an inhibitory antibody should only bind to Bb, or even better only to the C3bBb complex, so that a relatively low dose of the molecule be sufficient for complement inhibition. A blocking antibody, binding to free FB, which prevents the formation of the pro-convertase complex, is also a feasible option. In this case, also high doses would be required for optimal effect.

A set of small-molecule inhibitors are under development by Novartis against FB (CVF-Bb) for indications such as AMD and other complement-mediated diseases (159, 160). Neutralizing monoclonal antibodies against Bb by Novelmed Therapeutics are under development for various indications (161, 162). A monoclonal antibody to mouse FB has been shown to be protective in a mouse model of renal IRI (163). Other approaches using antisense oligonucleotides (164), or a phage-display selected cyclopeptides (165) are other feasible options to control AP activation through FB.

While FB is a promising target, so far, no therapeutic agent hit the market, or is in the advanced state in clinical trials. As with FD or MASP-3 inhibitors, bacterial infections manifest a potential risk when patients are treated with FB inhibitors.

### C3 and CVF

C3 is the central molecule of complement; the three activation routes are merged at the generation of C3b and continue together as the terminal phase (Figures 1 and 3). C3 circulates in the serum at high concentration (4–7 µM; 0.75–1.35 mg/mL). Native C3 is a 185 kDa protein containing 13 domains (Figure 2). C3 is composed of two chains, α and β. The core is built up of 8 domains belonging to the α2-macroglobulin family. The thioester domain carries a buried thioester bond, which is prone to suffer hydrolysis or other nucleophilic attack.

Primary C3 deficiencies were described in a few families over the world. Mutation in C3 gene caused impaired C3 synthesis or secretion, which produced a low C3 level in the blood. These individuals are extremely susceptible to recurrent pyogenic bacterial infections, especially to Gram-negative but also to Gram-positive bacteria (166). Moreover, C3 deficiency impairs maturation of immune cells (dendritic cells, memory B cells, certain T cells) (166, 167). Furthermore, SLE and various renal diseases were also observed; however, their mechanism is not fully understood. Secondary C3 deficiency is due to malfunctioning of the complement regulatory proteins, typically FI and FH (168).

**Figure 6:**
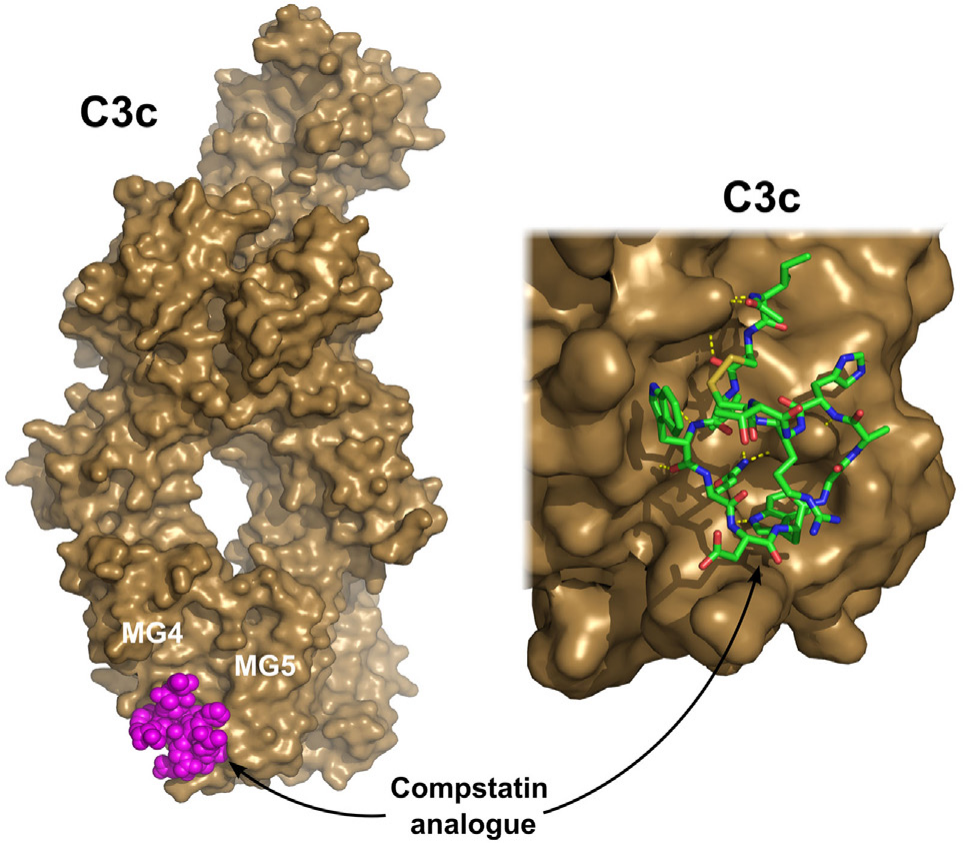
C3c in complex with a compstatin analog. Compstatin, a cyclic peptide, was developed by phage-display. Since its discovery, several modified compstatin analogs have been developed. Compstatin and its analogs bind to C3, C3b, or C3c between the MG4 and MG5 domains. Compstatin sterically prevents the C3-convertase (C3bBb) to access its substrate C3. The depicted structure was determined using the Ac-V4W/H9A-NH2 variant of the original peptide. The figure was prepared based on the structure by Janssen et al. (170) (PDB entry 2QKI). On the left, the whole structure is shown with C3c (brown) in surface representation and compstatin (magenta) with spheres. On the right, a close-up of the binding site is shown with compstatin represented by sticks. Hydrogen bonds are indicated by yellow dashed lines.

Complement activation can be blocked completely at the level of C3. On the other hand, C3 is the most abundant protein in the complement cascade; therefore, a large amount of an inhibitor would be needed to achieve a substantial effect. Compstatin, a promising complement-based therapeutic agent, was developed against C3 by phage-display using naïve library in 1996 (169). Compstatin is a cyclic peptide of 13 amino acids with a single disulfide bond. It blocks the access of the convertase to C3 through steric hindrance. Crystal structure with C3c showed that compstatin forms expansive H-bonds with its partner (170) (Figure 6). Neither complement regulator proteins nor other structurally related proteins (C4, C5) bind compstatin. In the past 20 years, the compstatin family has been constantly developed. New generations of compstatin analogs possess better pharmacokinetic and pharmacodynamic features. Compstatin derivatives were investigated in many complement-related animal diseases models and showed promising results (171). Just to mention a few, compstatins are efficient in primates in inflammatory diseases induced by cardiac surgery, cardiopulmonary bypass, or *E. coli* infection, in treatment of organ transplantation to reduce the possibility of xenograft rejection, and in sepsis. One of the compstatin derivatives (APL-2) already completed a Phase II clinical trial in treatment of AMD by Apellis Pharmaceuticals, and another one (AMY-101) started in 2017, the “first-in-human” clinical study against PNH by Amyndas Pharmaceuticals. Certainly, compstatins, as a peptide drug candidates, have their limitations especially considering oral administration. The rapid proteolytic degradation and poor biocompatibility make drug formulation challenging. On the other hand, the high specificity, the relatively low cost of production, and high variability gives the compstatin family members great potential to become widely used, effective, and safe complement therapeutics (171).

There are some other approaches to target complement activation through C3. Since 1970s, CVF is widely applied to deplete complement and to gain knowledge about its role in diseases. CVF is a structural and functional analog of C3, forms an AP convertase with Bb; however, it is resistant to the activity of FI and FH. Since decay of the convertase is abolished, C3 and C5 are rapidly exhausted from the blood. Nevertheless, CVF is immunogenic; therefore, it can be used only once to avoid antibody response. In the last decade, interesting results have been published about a chimeric protein, humanized CVF (172). It is a C3 derivative obtained by simply replacing the C-terminal part with the homologous sequence from CVF. This protein is safe and proved to be efficient in various animal disease models (AMD, collagen-induced arthritis, PNH, myasthenia gravis, etc.), and furthermore, no neutralizing antibody effect was detected in mice after prolonged usage (173).

### Properdin

The only known positive regulator of the AP is properdin, also referred to as factor P. Properdin circulates in the plasma at 20–125 nM (4–25 µg/mL) concentration as a cyclic polymeric glycoprotein. In contrast to most complement proteins, it is synthesized primarily by leukocytes and shows different activity depending on the type of producing cells. The properdin monomer comprises six complete and one truncated thrombospondin type 1 repeat (TSR) domain in tandem connection. The 53 kDa monomer is able to form dimers, trimers, and even tetramers in a head-to-tail arrangement. Physiologically, the most abundant form is the trimer; however, properdin shows tendency to self-aggregate into higher oligomers under conditions used for its preparation. It has an extremely high positive charge, hence it tends to bind *via* ionic interactions to polyanion structures.

Properdin has a significant and well-established role in the AP of complement by stabilizing the very labile C3bB and C3bBb complexes offering binding sites to C3b, and FB or Bb. Extending the half-life of the AP convertase by 5- to 10-fold is essential for the effective AP activity (174). Another role of properdin, serving as a PRM, was proposed about 10 years ago. A similar function was originally suggested by Pillemer, who discovered the “properdin pathway.” However, findings of this subject are controversial. Caution must be taken since repeated freezing and thawing resulted in highly polymerized, therefore, non-physiological, aggregated properdin, which binds non-specifically to surfaces. Experiments using unfractionated properdin could have led to physiologically not relevant observations (175). The binding abilities of properdin are also influenced by the contact surface and the presence of specific ligands. Experiments in properdin knock-out mice demonstrated that in the absence of properdin, bacterial LPS- and lipooligosaccharide-induced AP activation was absent, while zymosan or CVF-induced activation was only partially affected (79). Using compstatin and anti-FP antibody in ELISA assays, it has been shown that properdin does not attach directly to zymosan or *Escherichia coli* surfaces, but it contributes only to the stabilization of C3bBb complex (176). Recent studies showed that the binding of properdin to activating surfaces is always preceded by deposition of C3b (23), concluding that properdin can act as an initiator of AP only in a C3b-dependent manner.

Properdin deficiency especially in combination with the lack of other complement components (MBL, C2, etc.) causes unequivocal susceptibility to bacterial infections. Disorder in properdin and one of the late complement components (C5–C9) increases the risk of *Neisseria meningitidis* infection by 1,000- to 10,000-fold (28). Interestingly, the lack of IgG G2m(n) allotype in properdin-deficient persons also increased the susceptibility to meningococcal disease (177).

Therapeutic application of properdin emerged recently. In mouse model, it has been shown that a highly polymerized form of recombinant properdin gives protection against *N. meningitidis* and *Streptococcus pneumoniae*. A single low-dose treatment was enough to boost complement-activated lysis, which significantly reduced bacteremia and increased survival rates (178). We should note, however, that the recombinant properdin had histidine tag, which could influence its antimicrobial activity (179). Newly developed mouse monoclonal antibody against properdin was proved to be useful in sandwich-ELISA system to determine serum level of properdin in human samples (180). This antibody also successfully blocked AP activation in human sera.

The lack of properdin can efficiently abolish physiological or even unwanted AP activity. Inhibiting the AP through properdin can abate the amplification of deposited C3, hereby, the activity of the complement cascade. In contrast to C3, properdin is present at a relatively low concentration. Consequently, as an important regulator of the AP, it may turn out to be a promising therapeutic target to block complement activation. Novelmed is developing new drug candidates against the components of the AP, which do not interfere with the CP, as part of their effors, they evolved a new monoclonal antibody against the N-terminal fragment of properdin (181).

### Factor I

Factor I has an outstanding role in the control of the complement system. Along with its cofactors, FI belongs to the regulators of complement activation. Although it possesses a low catalytic activity on its own, FI can downregulate all activation routes by dismantling the central component of the complement cascade: C3b (and also C4b). FI contributes to the self-defense of host tissues against complement damage through the acceleration of the decay of fluid-phase and surface-bound C3b to iC3b. Degradation products of C3b initiate the cellular immune response *via* their interaction with various receptors on immune cells.

FI is an 88 kDa glycoprotein synthesized as a single polypeptide chain by hepatocytes. It is a trypsin-like SP consisting of five domains; some of them are common in the components of the terminal pathway. The heavy chain contains the first four domains: FI membrane attack complex domain, CD5-like domain, low-density lipoprotein receptor 1 and 2 (LDLr 1 and 2) domains, and a small section called d-region with unknown homology (Figure 2). The light chain, which is attached to the heavy chain by a disulfide bond consists of the catalytically active SP domain.

**Figure 7:**
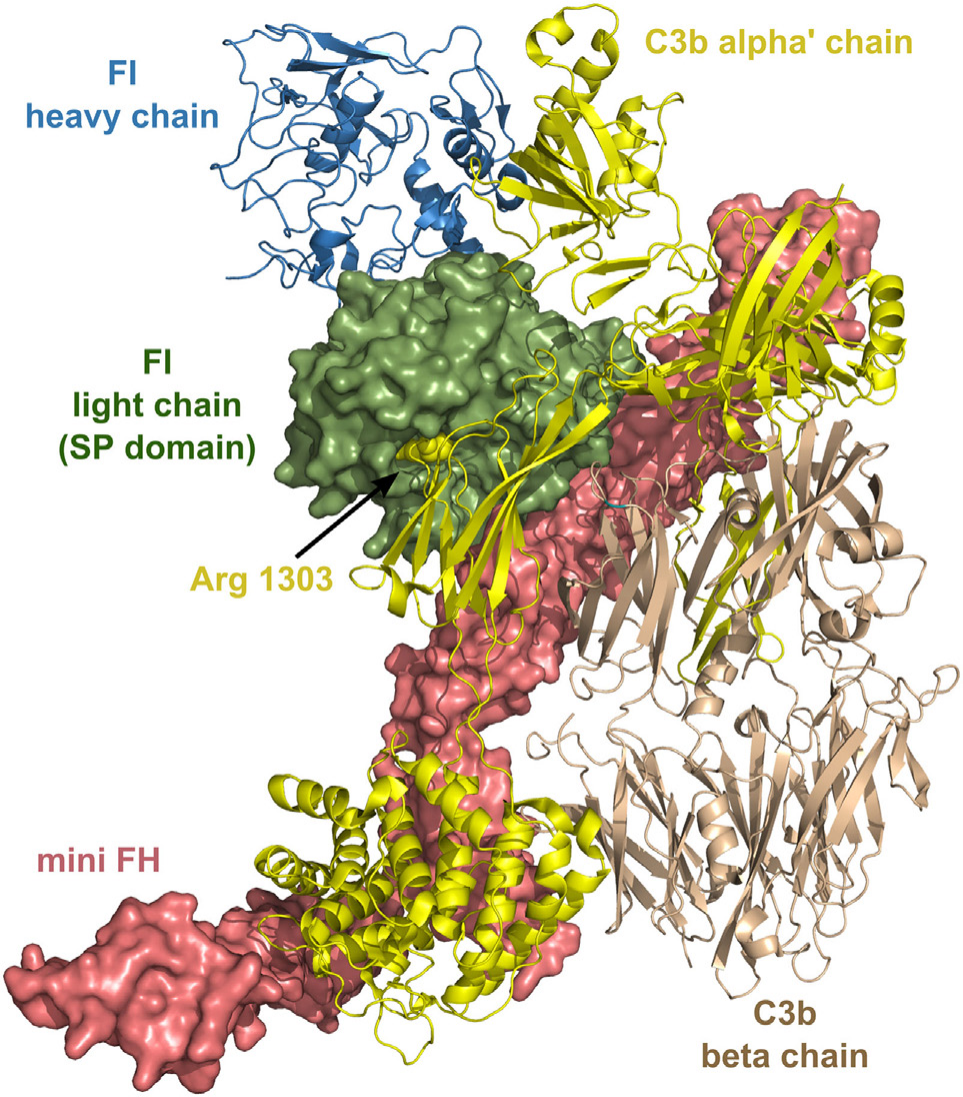
Structure of the complex of C3b, mini factor H (FH), and factor I (FI). Mini FH is a potential drug candidate. *In vitro*, it is more effective than full-length FH in accelerating the decay of C3b by FI. The structure shows extensive contacts between the three proteins. The figure was made based on the structure of ternary complex of C3b-mini FH-FI (S525A) (184) (PDB entry 5O32). The colors of the legends match the depicted protein chains. The P1 residue (Arg^1303^) of the primary cleavage site in C3b by FI is indicated by sphere representation. Mini FH and the light chain of FI are shown by surface representation, whereas C3b and the heavy chain of FI are shown by ribbon representation.

FI has several unusual characteristics: it circulates as an “active” enzyme in the blood and does not have an inhibitor (19). These and some other features of FI resemble that of two other proteases of complement, Factor D (182) and MASP-3. FI has an extremely low catalytic activity toward synthetic substrates and also toward free C3b and C4b. In order to cleave C3b and C4b efficiently, FI needs cofactors. C4b-binding protein (C4BP) and FH are soluble cofactors of FI, while MCP (CD46) and CR1 (CD31) are membrane-bound cofactors. Since no natural inhibitor of FI is known, it is regulated by other mechanisms. First of all, the type of the substrate and also the type of its cofactor influences the activity of FI, and also the cleavage site on C3b or C4b and their degradation products. Second, structural data has proven recently that many crucial loops of the SP domain are disordered without the interacting partners (183). As the ternary complex of C3b-FH-FI is formed, ligand binding induces stabilization of the SP domain and, therefore, FI obtains full proteolytic activity. After the cleavage of the first bond in C3b (Figure 7), the substrates rearranges and the second or third cleavage site becomes accessible, while the SP domain of FI endures only minor movements (184).

According to its important role in complement regulation, the absence of FI causes dangerous, even life-threatening conditions. Due to the lack of decay acceleration, increased amounts of C3b lead to uncontrolled AP activation. The more C3b molecules are present, the more C3 convertases are generated, which results in the rapid exhaustion of C3 from the plasma. Individuals with FI deficiency are prone to suffer from recurring bacterial infections, severe kidney diseases, and most of all AMD (185, 186). Recent studies show that identifying rare CR1 variants in combination with low serum level of FI can enable therapist to find patients, who are the most likely candidates to develop AMD (187). FI deficiency is often associated with aHUS. Symptoms frequently appear in early childhood after a severe infection or in young females shortly after pregnancy. Large international cohorts have been established to characterize all genetic variants and clinical outcomes. The prognosis of FI-associated aHUS is quite poor, in half of the cases, end-stage renal failure developed rapidly. Treatment with eculizumab, which is the major therapeutic for aHUS, resulted in partial remission in patients having FI-associated aHUS (188).

Another aspect that makes FI a potential drug candidate is the phenomenon of multiple polymorphisms in complement components that can affect the delicate balance between activation and regulation of an individual’s complement system. The inherited repertoire of the complement gene variants was dubbed complotype (189). Some variant alleles can result a more reactive complement, which usually appears in the increased activity of C3b feedback cycle. Hyperactive complotypes raise significantly the risk of complement-related diseases at later age. Lower serum level of FI compared to FH (along with FH-related proteins) is advantageous to produce an effect. Moreover, the administration of FI in the presence of cofactor CR1 also enhances the conversion of inflammatory product iC3b to C3dg (190). Experimental data prove that increasing the amount of FI in serum of different complotypes can convert higher-risk to lower-risk activity. The extra amount of FI needed is approximately 50% of normal level, which would be a useful therapy in such patients (191). Comprehensive characterization of the complement regulatory genes in patients already suffering from complement-related disorders would enhance developments of personal and successful therapies.

### Factor H, FH-Like, and FH-Related Proteins

Factor H (FH) is the major regulator of the AP. It is a fluid-phase molecule; however, it can bind to surface-deposited C3b and regulate the AP C3 convertase by several ways. By binding to C3b, it can prevent the capture of FB, consequently, the formation of the pro-convertase (C3bB). It has also a convertase decay-accelerating activity by facilitating the irreversible dissociation of the C3bBb complex. Probably, the most important function of FH is the cofactor activity, which is necessary for the FI-mediated cleavage of C3b to iC3b, through which it prevents the build-up of the amplification feedback loop of the AP. FH is a glycoprotein of 115 kDa and it consists of 20 CCP (aka short consensus repeat or sushi) domains (Figure 2). These domains, which are about 60 residues in length and contain two highly conserved disulfide bonds, are widespread among the complement proteins. Many complement regulatory proteins, such as FH and the FH-related proteins, CR1, CR2, MCP, DAF, C4BP, are composed predominantly or exclusively of these repeating structural motifs. The four N-terminal CCP domains of FH are responsible for the convertase decay-accelerating and cofactor activity. The other CCP domains take place in the interaction with different ligands. The C-terminal CCP 19–20 domains are indispensable for binding to self-surface deposited C3b. According to the current knowledge, FH recognizes the juxtaposition of C3b and carbohydrates containing sialic acid or glycosaminoglycan on the surface and binds strongly through the CCP 19–20 domains. The other domains may contribute to the binding to several ligands (e.g., heparin/CCP 7), but they are not indispensable for the function of FH. Based on this knowledge, minimal-size FH molecules were designed by combining the N- and C-terminal regions. Two constructs contain only six domains (CCP1-4 and 19-20) (192, 193). These mini FH molecules (Figure 7) showed more effective complement inhibition in different assay systems than the full-length FH molecule (192). Another, slightly extended construct containing CCP1–5 and 18–20 domains effectively inhibited complement activation *in vivo* and reduced abnormal glomerular C3 deposition in a FH-deficient mouse model of C3 glomerulopathy (194). Recently, a monoclonal anti-FH antibody has been found that could inhibit AP activation by potentiating FH (195). This potentiating antibody increases the affinity of FH for C3b and facilitates the degradation of the convertase by FH. There is an alternative splice product of the FH gene, which consists of only the CCP1-7 domains plus a four-amino-acid long C-terminus (196). This FH-like protein 1 (FHL-1) has complement-inhibitory activity, and it may have important function in the periphery. It is supposed that FHL-1 is able to penetrate through the Bruch’s membrane beneath the retinal pigment epithelial cells in the eye, while FH cannot. In this way, FHL-1 may have a crucial role in the protection of retinal cells against complement-mediated attack and prevention of the development of AMD (197). Besides FH and FHL-1, there are five FH-related proteins (FHR) in the human serum. These proteins are encoded by separate genes situated next to the FH gene, and these genes arouse very probably through partial gene duplications. These proteins are shorter than FH and usually consist of CCP domains homologs to CCP6-9 and CCP18-20 of FH. Since the FHRs lack the complement regulatory CCP1-4 domains, their physiological relevance was underestimated at the time of their discovery (198). Since then, increasing number of evidences have been accumulated demonstrating the physiological role of FHRs, although this area is still controversial. Since the FHRs contain domains sharing high sequence identity with CCP18-20 of FH, these proteins can bind to ligands of FH (e.g., C3b, heparin, CRP). However, these molecules cannot efficiently inhibit the AP since they lack the N-terminal regulatory domains of FH (CCP1-4).

FHR-1 was reported to enhance, rather than to inhibit complement activation through binding to CRP (199). This phenomenon could explain the protective effect of FHR-1 deficiency in AMD (200). FHR-4 was able to facilitate AP and CP activation by binding to C3b and CRP, respectively (201). FHR-5 was also shown to promote complement activation by binding to pentraxin 3 (PTX3) and extracellular matrix and by enhancing C1q deposition (202). It is also possible that FHRs compete with FH on the surface of bacteria, thereby compromising the ability of the microorganism to evade complement-mediated attack (203). It has been demonstrated that FHR-3 acts as a decoy, being captured by *N. meningitidis* cells instead of FH (204). The level of protection against *N. meningitidis* infection may depend on the FH/FHR-3 ratio in the serum. In general, the serum concentration of FH and the FHRs, and their affinity to various ligands may be a key factor in the fine tuning of complement-mediated opsonization and inflammation. If the delicate balance between FH and FHRs is disturbed due to genetic variations, or the amount and the composition of the ligands changes in the course of a disease (infection, oxidative stress), improper complement activation can take place resulting in self-tissue damage.

## Concluding Remarks

The complement system was an appealing drug target even in the 1970s. However, the early drug development efforts failed mainly because of two reasons. The first reason was the lack of specificity of the anticomplement compounds. At that time, there was no technology to design or select highly specific agents against the individual complement components. The advance of structure-based and fragment-based drug design approaches made possible to generate selective and efficient small-molecule drugs. In addition to that, the modern biotechnological methods have provided highly specific biologics [monoclonal antibodies, recombinant proteins, nucleic-acid aptamers (205, 206), etc.] developed for anticomplement therapy. The most successful anticomplement drug so far, eculizumab, is a monoclonal antibody, and many antibodies are in preclinical or clinical phase in the pipeline. The second reason, which hindered the introduction of anticomplement drugs in the clinical practice in the past, was the insufficient knowledge about the mechanism of action of complement in both health and disease. In the recent years, the mechanism of activation and regulation of the LP and AP has been revealed in more detail, and we got insight into the cross-talks between the individual pathways inside the complement system and also the cross-talks between the complement and other proteolytic cascade systems (e.g., coagulation). New discoveries have also been made about the role of complement in the regulation of the adaptive immune system. Based on all of the above mentioned scientific and technical advances, essentially, all components of the LP and AP became targets of drug development (Table 1). It is likely that new drugs with more efficiency and less adverse effect will be approved for treating complement-related disorders in the near future.

**Table 1:** Potential drug targets of the complement system discussed in this review.

Protein	Role in complement activation	Expected effects of inhibition	Potential diseases	Type of the drug candidate molecules	Reference
C1q	Pattern recognition molecule (PRM) of the classical pathway (CP)	Blocking the CP	Neurodegenerative diseases	Antibody, peptide	(84, 86–88)

MBL, ficolins	PRM of the lectin pathway (LP)	Blocking the LP	Ischemia–reperfusion injury (IRI)	Antibody	(84, 85)

MASP-1	LP initiation, boosting the alternative pathway (AP)	Attenuation of the LP and LPS-driven AP	IRI	Protein, small protein	(113, 121, 122)

MASP-2	LP initiation	Attenuation of the LP	IRI, renal diseases, atypical hemolytic uremic syndrome (aHUS)	Antibody, small protein	(121, 122, 129–132)

MASP-3	AP pre-initiation	Attenuation of the AP	AP-driven diseases	Antibody, small protein	(75, 138)

FD	AP initiation	Attenuation of the AP	Age-related macular degeneration (AMD), renal diseases	Antibody, small molecule	(120, 150, 154)

FB	Driving the AP	Attenuation of the AP	AMD, renal diseases	Small molecule, antibody	(158–165)

C3/C3b	Component of the AP C3 convertase and both C5 convertases	Blocking of AP and TP	AMD, paroxysmal nocturnal hemoglobinuria (PNH), renal diseases, transplantation	Peptide, protein	(169, 171–173)

Properdin	AP C3/C5 convertase stabilization	Attenuation of the AP	AMD, PNH	Antibody	(160, 181)

FI	Regulation of all pathways *via* degradation of C3b and C4b	NA	AMD, aHUS	Protein (FI for replacement therapy)	(191)

FH	AP regulation	NA	AMD, aHUS, transplantation	Antibody, proteins (rec. FH constructs for replacement therapy)	(192–195)

*NA, not applicable, because inhibition of negative regulators is generally not desirable; however, potentiation could be a feasible approach (195)*.

## Author Contributions

All authors contributed equally to this article.

## Conflict of Interest Statement

The authors declare that the research was conducted in the absence of any commercial or financial relationships that could be construed as a potential conflict of interest.

## Abbreviations

3MC syndrome, Malpuech–Michels–Mingarelli–Carnevale syndrome; aHUS, atypical hemolytic uremic syndrome; AMD, age-related macular degeneration; AP, alternative pathway; C4BP, C4b-binding protein; CARPA, complement activation-related pseudoallergy; CCP, complement control protein; CP, classical pathway; CNS, central nervous system; CR1, complement receptor 1; CR2, complement receptor 2; CRP, C-reactive protein; CUB, C1r/C1s, the sea urchin protein Uegf, and the human bone morphogenetic protein 1; CVF, cobra venom factor; EGF, epidermal growth factor; DAF, decay-accelerating factor; DAMP, damage-associated molecular pattern; FHL, FH-like protein; FHR, FH-related protein; FIMAC, FI membrane attack complex; GPI, glycosyl phosphatidylinositol; HAE, hereditary angioedema; HUVEC, human umbilical vein endothelial cell; LDLr, low-density lipoprotein receptor; LP, lectin pathway; MAC, membrane attack complex; MBL, mannose-binding lectin; MASP, MBL-associated serine protease; MCP, membrane cofactor protein; IGFBP-5, insulin-like growth factor-binding protein 5; IRI, ischemia–reperfusion injury; LPS, lipopolysaccharide; PAMP, pathogen-associated molecular pattern; PNH, paroxysmal nocturnal hemoglobinuria; PRM, pattern recognition molecule; SIRS, systemic inflammatory response syndrome; SP domain, serine protease domain; SLE, systemic lupus erythematosus; TAFI, thrombin-activatable fibrinolysis inhibitor; TSR, thrombospondin type repeat; TCC, terminal complement complex; VWA, Von Willebrand factor type-A.
